# Atomic Diffusion and Crystal Structure Evolution at the Fe-Ti Interface: Molecular Dynamics Simulations

**DOI:** 10.3390/ma15186302

**Published:** 2022-09-11

**Authors:** Guojin Xiang, Xu Luo, Tianxu Cao, Ankang Zhang, Hui Yu

**Affiliations:** 1 School of Mechanical Engineering, Yanshan University, Qinhuangdao 066000, China; 2 Pangang Group Research Institute Co., Ltd., Panzhihua 617000, China

**Keywords:** diffusion bonding, molecular dynamics, diffusion mechanism, shear strain, diffusion coefficient

## Abstract

The diffusion bonding method is one of the most essential manufacturing technologies for Ti-steel composite plates. In this paper, the atomic diffusion behavior at the Fe-Ti interface during the bonding process of Ti-steel composite plates is studied using classical diffusion theory and molecular dynamics (MD) simulation. Henceforth, the diffusion mechanism of Fe and Ti atoms at the bonding interface is obtained at the atomic scale. The results show that Fe and Ti atoms diffused deeply into each other during the diffusion process. This behavior consequently increased the thickness of the diffusion layer. Moreover, the diffusion quantity of Fe atoms to the Ti side was much greater than that of Ti atoms to the Fe side. Large plastic deformation and shear strain occurred at the diffusion interface during diffusion. The crystal structure of the diffusion zone was damaged and defects were generated, which was beneficial to the diffusion behavior of the interface atoms. As the diffusion time and temperature increased, the shear strain of the atoms at the interface also increased. Furthermore, there is a relationship between the mutual diffusion coefficient and the temperature. Subsequently, after the diffusion temperature was raised, the mutual diffusion coefficient and atomic disorder (Fe atom and Ti atom) increased accordingly.

## 1. Introduction

The Ti-steel composite plate is a kind of composite metal plate that applies heating, pressure, or two types of combination procedures to create strong metallurgical bonding at the interface between the titanium plate and steel plate. In some circumstances, the combination of titanium and steel produces components with both strong corrosion resistance and the price advantages of titanium and steel, thus, effectively utilizing the complementary advantages of titanium and steel in performance and economy. Researchers have conducted a lot of research work to solve the problem of insufficient bonding strength of the composite plates, which is caused by bonding interface defects in Ti-steel composite plates. According to a large number of studies, the intermediate compounds formed on the composite interface are the primary motivation for the interface bonding limitation of the composite plate, which leads to the formation of interface defects. The more kinds of interfacial compounds, the greater the influence on interfacial bonding strength [1,2].

Jiang et al. [3] performed heat treatment on the Ti-steel composite plate produced by the explosive composite method. It was observed that the major compound generated at the composite interface at 850 °C was TiC. At 950 °C, the main products were Fe_2_Ti, FeTi, and a trace of TiC. Momono et al. [4] investigated the influence of C element mass fraction on Ti-steel diffusion welding performance. When the steel with 0.01 % C element was heated at temperatures over 900 °C, FeTi and Fe_2_Ti compounds were formed at the composite interface, reducing composite strength. TiC, FeTi, and Fe_2_Ti compounds were produced at the composite interface in the steel with a 0.19 % mass fraction of C when the temperature exceeded 900 °C and the composite strength dropped. The grain orientation of titanium has a great influence on the interfacial reaction behavior of titanium and steel. When the basal plane of a titanium cell is parallel to the Fe/Ti interface, it has a strong inhibitory impact on the diffusion of C atoms and the TiC layer is thin, according to Li et al. [5] The TiC layer is thicker when the titanium cell base surface is perpendicular to the titanium/steel interface. This is owing to the anisotropy of the gap carbon atom transport in titanium cells.

Molecular dynamics (MD) is a powerful approach for simulating molecular systems using Newtonian mechanics. It can explain some macroscopic properties of matter and conduct an experiment at the micro level, which is significant for revealing the development of atomic-scale structures. Nowadays, MD simulation has become an effective tool for studying the interface diffusion bonding process.

Firstly, Leo Miglio et al. [6] investigated the mathematical simulation of Si diffusion in TiS_2_ compounds and discovered that the diffusion of Si in this compound was mostly owing to its low formation energy. Since then, numerous researchers have investigated the diffusion phenomena by MD simulation. Chen et al. [7] simulated the formation process of the Cu-Ag diffusion bond using the MD method and discovered that the thickness of the interface area is mainly dictated by stress. In the process of diffusion bonding, the interface region is transformed into an amorphous form. However, it often transitions from an amorphous to a crystal structure when it cooled to room temperature. Chen et al. [8] used MD simulation to investigate atomic diffusion during explosive welding of Ni_50_Ti_50_-Cu (at. %). The geometric similarity of concentration distribution curves at different points during diffusion was applied to compute the distribution of atomic concentration at any time throughout the explosive welding process and the simulation results are virtually identical to the experimental results. Similarly, Xiu et al. [9] applied MD to simulate W/Cu diffusion bonding. The atomic diffusion behavior of W and Cu atoms was investigated in their study and the diffusion coefficient and radial distribution function (RDF) were also calculated. The diffusion mechanism of the W/Cu diffusion bond is as follows: when the temperature and diffusion time rise, the thickness of the diffusion layer and atomic disorder increase as W and Cu atoms propagate along the crystal defect surface. Solid-state wetness (SSW) happens not only at the micron scale but also at the nanoscale, according to research by M. Samsonov et al. [10], who simulated the spread of solid copper (Cu) and gold (Au) nanoparticles over the same metal (100) surface. The temperature effect is stronger than the pressure effect during diffusion, according to research by Zhang et al. [11], which looked at the effects of temperature, pressure, and surface roughness on the diffusion welding of stainless steel and pure Ni. By simulating self-diffusion along the screw dislocation core in the metals aluminum, nickel, copper, and silver, Soltani et al. [12] simulated the effect of screw dislocation on boosting self-diffusion for each metal mentioned above. In a nanostructured Cu/Ag system, Béjaud et al. [13] investigated the interaction between interface and deformation twins and precisely analyzed the effects of misfit dislocation on twin nucleation and thickening in a face-centered cubic structure. Yang et al. [14] investigated the diffusion behavior of Al and Cu atoms in ultrasonic welding using MD modeling. Their findings revealed that asymmetric diffusion occurred at the Al/Cu interface during the ultrasonic welding (UW)process. Simultaneously, the recovery of disordered Al blocks at low temperatures was observed and the thickness of the diffusion layer increased as welding time increased. Because of the significant trapping effect of the grain boundaries in nanocrystalline Fe, Zhou et al.’s study [15] on the dependency of the hydrogen diffusion coefficient on grain size revealed that smaller grain sizes correspond to reduced hydrogen diffusion coefficients. The effects of temperature and pressure on atomic interdiffusion along the direction perpendicular to the Cu/Al solid–liquid interface were revealed by Mao et al. [16] and found that while the thickness of the diffusion layer exhibits a parabolic connection with diffusion time, the Cu atoms’ depth of diffusion exhibits a linear relationship with system temperatures. Using the MD approach, Chen et al. [17] investigated the influence of temperature on the diffusion rate and mechanical characteristics of the nano-scale TiAl/Ti_3_Al interface. The elastic modulus, yield strength, and flow stress all reduced as the temperature increased from 1273 K to 1473 K. Wang et al. [18] investigated the atomic diffusion behavior of the Mo/Au interface using classical diffusion theory and MD simulation and the mutual diffusion coefficient and radial distribution function of Mo and Au atoms were obtained. In a recycled asphalt mixture, Zhan et al. [19] described the behavior of new asphalt and aged asphalt diffusion, which revealed that the diffusion direction was mostly from virgin asphalt to aged asphalt and that the diffusion efficiency improved with temperature. Zurhelle et al. [20] studied the oxygen atoms’ diffusion characteristics in platinum atoms with extended defects and their results demonstrated that platinum vacancies prevent oxygen atom diffusion across the platinum lattice by trapping oxygen atoms. Amorphization of Cu atoms during diffusion was noted in this study by Zhang et al. [21], who simulated the diffusion process and atomic structure of the interface between metallic glass Cu_50_Zr_50_ and crystalline Cu.

In this paper, MD simulation was used to study the atomic diffusion behavior and crystal structure evolution at the the Fe-Ti interface in the diffusion bonding process of the Ti-steel composite plates and classical diffusion theory. The diffusion process of Fe-Ti interface was characterized through the calculation of profiles for atomic trajectory, atomic concentration distribution, diffusion layer thickness, atomic shear strain, radial distribution function (RDF), mean square displacement (MSD), and diffusion coefficient. The relationship between the calculation profiles and diffusion temperature was also presented, which will benefit our understanding of the atomic diffusion mechanism in the Fe-Ti interface.

## 2. Simulation Method

### 2.1. Potential Function

Potential function describes the interaction of atoms or molecules, also known as a force field. The correctness of the potential function has a significant impact on the dependability of MD simulation results. Daw and Baskes [22] developed the embedded atom method (EAM) based on density functional theory and effective medium theory in 1983. It could solve the inadequacies in the two-body potential model that are incompatible with particle interaction in metal systems. Furthermore, in order to implement the EAM potential to covalent bond materials, the non-spherical symmetric distribution of electrons should be considered. Baskes et al. [23] developed a modified embedded atom method (MEAM). The potential function of MEAM is represented as follows:

(1) $$E=\sum_{i}\{F_{i}(\vec{\rho_{i}})+\frac{1}{2}\sum_{i\neq j}S_{ij}\phi_{ij}(R_{ij})\}$$

(2) $$F_{i}(\vec{\rho })=A_{i}E_{i}^{0}\vec{\rho_{i}}\ln\vec{\rho_{i}}$$

where *E* is the total energy in the system; *F_i_* is the embedding function for an atom *i* embedded in a background electron density *ρ_i_*; and *S_ij_* and *ϕ_ij_ (R_ij_)* are the screening function and the pair interaction between atoms *i* and *j* separated by a distance *R_ij_*; $E_{i}^{0}$ is the binding energy of atom *I*; and *A_i_* is the structure parameter.

The embedded atomic method (MEAM) interatomic potential of the Fe-Ti-C binary alloy system was developed by Kim et al. [24]. The MEAM potentials were validated in the literature by Prasanthi et al. [25]. At an equilibrium distance of 2.879 Å, they calculated that the cohesive energy for the Fe–Ti system was 4.360 eV, which was quite compatible with MEAM potentials. As well, the calculated bulk moduli of Fe (153 GPa) and Ti (110 GPa) were in good agreement with the experimentally measured values of 166 GPa for "Armco Iron" and 108 GPa for Ti [26,27].

In addition, by examining the overall structural consistency of atoms in the system before and after the relaxation process, it is demonstrated that utilizing this potential function for relaxation yields a relatively stable starting model, which is adequate for the simulation system.

### 2.2. Simulation Model

**Figure 1:**
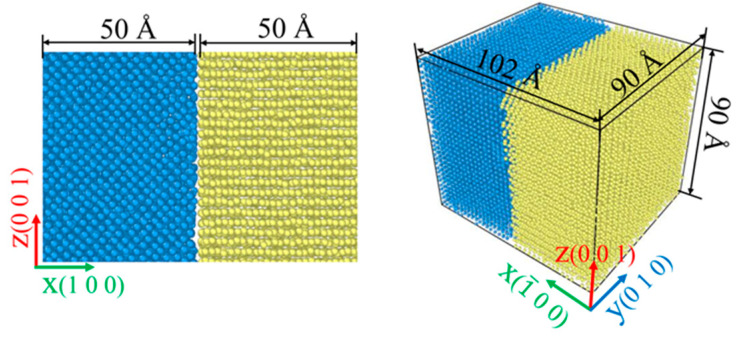
Simulation model.

The lattice type of Fe and Ti at home temperature is a body-centered cubic and hexagonal close-packed structure, respectively. The lattice constants of Fe and Ti are 0.286 nm and 0.295 nm, respectively. Where the size of Fe is 90 Å × 90 Å × 50 Å and the size of Ti is 90 Å × 90 Å × 50 Å, as depicted in Figure 1. Among them, the blue atoms are Fe atoms and the yellow atoms are Ti atoms. The total numbers of the Fe and Ti atoms in the model were 33,597 and 22,971, respectively. There is an initial gap of 2 Å between the Fe and Ti samples, which aims to reduce the strong interaction force between the two atoms at the interface, causing the samples to fit better.

The large-scale atomic molecular massively parallel simulator (LAMMPS) program is used in this paper [28]. To begin with, the two models are completely relaxed by the normal pressure and temperature (NPT) ensemble with a goal temperature at the time of 500 ps to reach a rather stable state. Then, they are merged in the y-z surface and the periodic boundary conditions in the y and z directions are applied, while the shrinking boundary conditions are applied in the x-direction. The ensemble is set to NVT (N: number of particles; V: volume; T: temperature) and the Nosé–Hoover method is used to maintain a constant system temperature of 1123 K and no external pressure is applied to the whole system. The timestep is 0.001 ps and the initial velocity of atoms follows the Maxwell distribution.

## 3. Result and Discussion

### 3.1. Interface Diffusion Behavior

**Figure 2:**
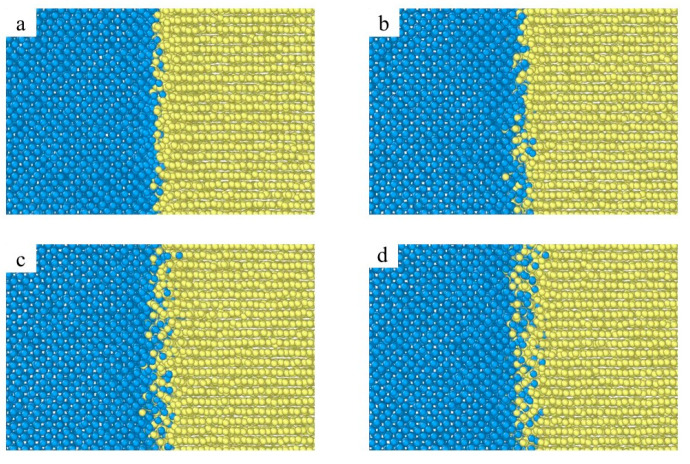
Atomic diffusion behavior of Fe/Ti diffusion interface at the temperature T = 1123 K ((**a**) 0.001 ns; (**b**) 0.5 ns; (**c**) 1.5 ns; (**d**) 2ns).

**Figure 3:**
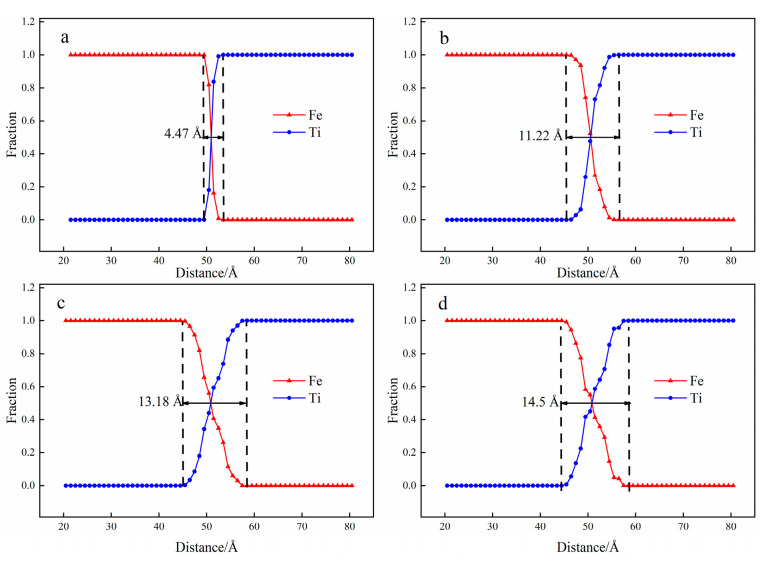
Atomic concentration distribution at the temperature T = 1123 K ((**a**) 0.001 ns; (**b**) 0.5 ns; (**c**) 1.5 ns; (**d**) 2 ns).

Figure 2 shows the atomic diffusion behavior of the Fe/Ti diffusion interface at the temperature T = 1123 K, (a) 0.001 ns; (b) 0.5 ns; (c) 1.5 ns; (d) 2 ns. The diffusion area between Fe and Ti is defined as the region between the farthest diffused Fe atom (i.e., the Fe atom with the largest x coordinate) and the farthest diffused Ti atom (i.e., the Ti atom with the smallest x coordinate). A distinct interface was produced at the time of t = 0.001 ns and t = 0.5 ns, as shown in Figure 2a,b, in which a tiny quantity of Fe atoms diffused into the Ti layer and a few Ti atoms diffused into the Fe layers. As the diffusion time increased to t = 1.5 ns and t = 2 ns, more Fe atoms diffused into the other side and the diffusion area became thicker, as shown in Figure 2c,d. Additionally, the interface between the Fe layer and Ti layer began to diminish as the diffusion time increased. This phenomenon can be explained as follows: binding energy was produced as the Fe layer contacted with the Ti layer, which enhanced the interaction and movement between Fe and Ti atoms. Figure 3 illustrates the atomic concentration distribution at different diffusion times. As the time increased from t = 0.001 ns to t = 1.5 ns, the thickness of the diffusion area increased from 4.47 Å to 11.22 Å. While the diffusion area just increased to 1.32 Å as the time rose from t = 1.5 ns to t = 2 ns, as shown in Figure 3a,d, whereas the number of Fe atoms (or Ti atoms) entering the titanium lattice (or Fe lattice) is still increasing as the time increases from t = 1.5 ns to t = 2 ns, as shown in Figure 3c,d. This result indicates that when the diffusion time is sufficient, the thickness of the diffusion area will no longer increase, but atoms will always enter the diffusion region.

**Figure 4:**
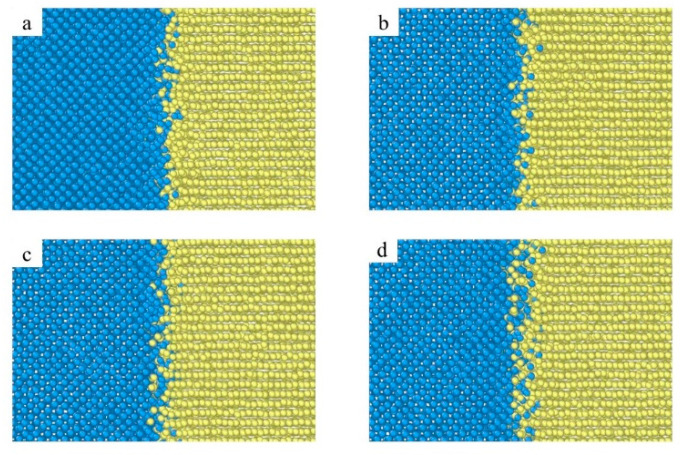
Atomic diffusion behavior of Fe/Ti diffusion interface at the time t = 2 ns ((**a**) 973 K; (**b**) 1023 K; (**c**) 1073 K; (**d**) 1123 K).

Figure 4 shows the atomic diffusion behavior of the Fe/Ti diffusion interface at different temperatures and the atom diffusion behavior is temperature dependent. The number of diffusion atoms rises with the increase in diffusion temperature. At a temperature of 973 K, a few Fe atoms diffuse into the Ti layer and nearly no Ti atoms diffuse into the Fe layer, as shown in Figure 3a. A growing number of atoms diffuse into the other side as the diffusion temperature increases and the diffusion area becomes thicker, as shown in Figure 3b,d. Furthermore, the overall number of Ti atoms that diffused and the diffusion depth are significantly smaller than those of Fe atoms in the titanium lattice, indicating asymmetric diffusion. 

**Figure 5:**
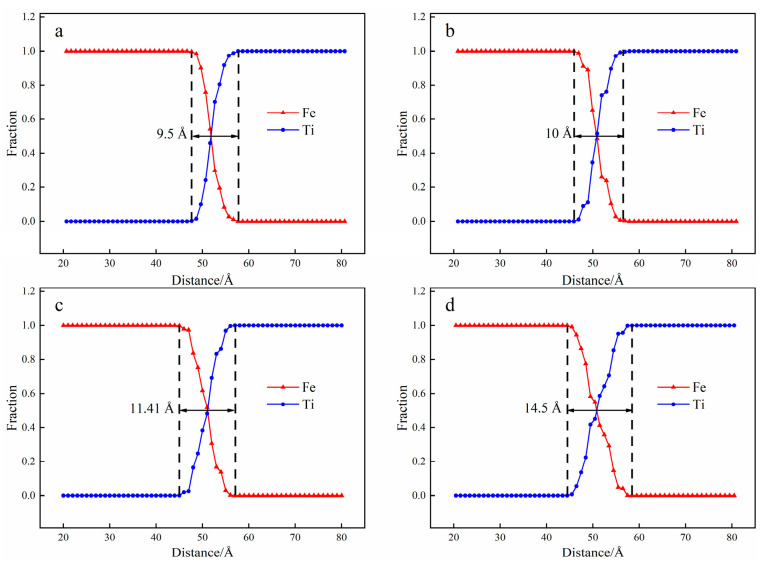
Atomic concentration distribution at the time t = 2 ns ((**a**) 973 K; (**b**) 1023 K; (**c**) 1073 K; (**d**) 1123 K).

The atomic concentration distribution curves at different temperatures are depicted in Figure 5. The thickness of the diffusion and the number of atoms in the diffusion region increase with the rise in the diffusion temperature. The thickness of the diffusion zone increases from 9.5 Å to 10 Å as the diffusion temperature increases from 973 K to 1023 K. Similarly, when the diffusion temperature is 1073 K and 1123 K, the thickness of the diffusion zone increases to 11.41 Å and 14.5 Å, respectively. Further, the higher the temperature, the more pronounced the effect. All in all, diffusion temperature plays an effective role in promoting interface diffusion between Fe and Ti layers.

**Figure 6:**
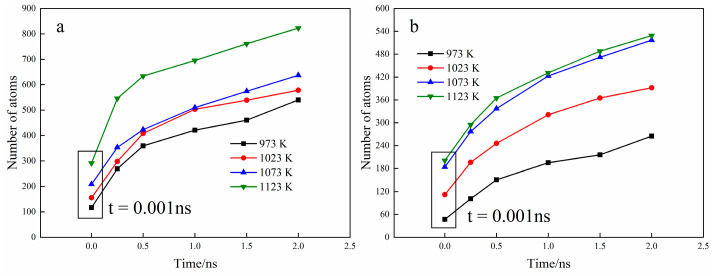
Number of diffused atoms with specific time and temperature ((**a**) Fe atoms; (**b**) Ti atoms).

The number of diffused atoms was counted by the means of the “selection” function in the open visualization tool (OVITO) [29]. Figure 6 shows the number of diffused atoms of Fe and Ti after each specific time and temperature and the number of diffusion atoms increases with the increase in temperature and diffusion time and the curve gradually becomes gentle over time, which showed a linear relationship with the diffusion time after t = 0.5 ns. In addition, the number of diffused Fe atoms is always greater than that of Ti atoms, which is consistent with the previous analysis results.

### 3.2. Crystal Structure Analysis

**Figure 7:**
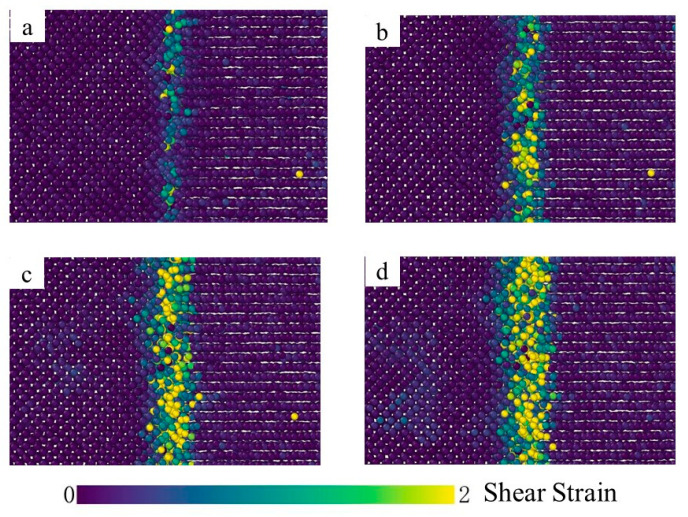
Shear strain distribution at the temperature T = 1123 K ((**a**) 0.001 ns; (**b**) 0.5 ns; (**c**) 1.5 ns; (**d**) 2 ns).

**Figure 8:**
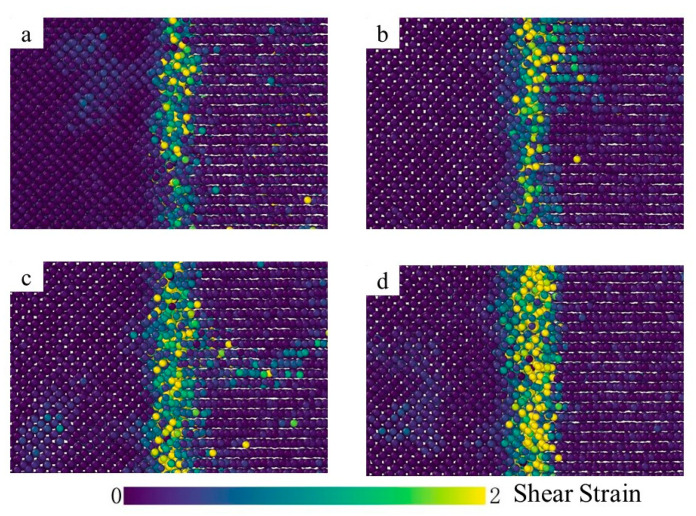
Shear strain at the time t = 2 ns ((**a**) 973 K; (**b**) 1023 K; (**c**) 1073 K; (**d**) 1123 K).

Figure 7 depicts the shear strain distribution at different diffusion times and a narrow shear plastic deformation zone was observed at the bonding interface. At the time of t = 0.001 ns, a limited number of atoms are influenced by the plastic deformation and the value of the shear strain is negligible, as depicted in Figure 7a. Furthermore, from t = 0.001 ns to t = 2 ns, the shear strain gradually increases and the plastic deformation band spreads to both sides, as shown in Figure 7a–d, indicating the deformation of the interface area during interface bonding. As a result, a large number of Fe and Ti atoms migrate from the original equilibrium position to a new position on both sides of the adjacent interface, so that the vacancies appear near the binding interface and the vacancy concentration increases continuously. Since the activation energy of high-density diffusion is much lower than that of lattice diffusion, the vacancy can be considered as the best place for large-scale diffusion of dissimilar atoms, so mutual diffusion between Fe and Ti atoms occurs when the vacancy on the iron side (or Ti side) reaches a new equilibrium. It can also be found in Figure 8 that temperature has a significant effect on the atomic shear strain in the diffusion area. The atomic shear strain in the diffusion zone rises with the increase in temperature and the effect range expands with the increase in diffusion zone thickness.

Radial distribution function (RDF) is an effective method for describing system structure, which can characterize the disorder degree of the structure [30]. The RDF refers to the probability of finding another atom at a distance r from one atom, which can be used to represent information, such as the relationship between atoms and the interaction intensity, so as to study the atomic structure.

**Figure 9:**
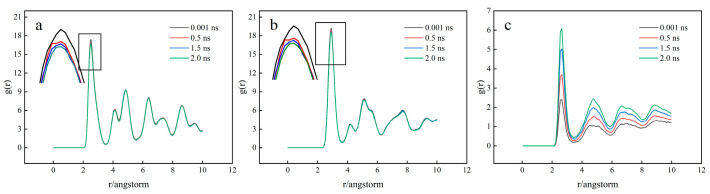
RDF curves of Fe and Ti atoms at T = 1123 K ((**a**) Fe-Fe; (**b**) Ti-Ti; (**c**) Fe-Ti).

**Figure 10:**
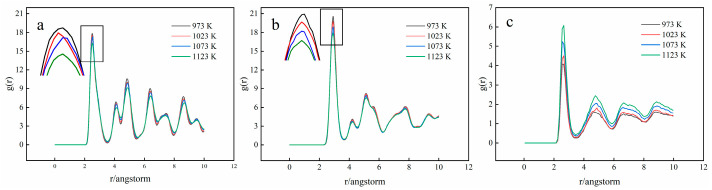
RDF curves of Fe and Ti atoms at t = 2 ns ((**a**) Fe-Fe; (**b**) Ti-Ti; (**c**) Fe-Ti).

Figure 9 and Figure 10 show the RDF curves (g(r)) of Fe and Ti atoms at different diffusion times and diffusion temperatures, respectively. At different diffusion times and temperatures, the RDF curves of Fe and Ti atoms are essentially the same. There is only one main peak, representing the binding strength between the first nearest neighbor atoms of the central atom. Its sharp shape indicates that the number density of atoms in this radius range is much higher than the average density and the binding strength between the central atom and the nearest neighbor atom is also relatively large. As presented in Figure 9a, the main peak of g(rFe-Fe) (the RDF curve of the Fe-Fe atom) appears at r = 2.55 Å, suggesting that the nearest atoms of Fe atoms appear at r = 2.55 Å and the bond length of Fe-Fe can be obtained at 2.55 Å. Furthermore, Figure 9b depicts that the nearest atom of Ti atom could be found at r = 2.93 Å and the Ti-Ti bond length can be gained at 2.93 Å. Similarly, the bond length of Fe-Ti can be calculated at 2.65 Å, as shown in Figure 9c, which is consistent with the previous simulation results [31]. Furthermore, it could be found that the value of r(Fe-Ti) (the bond length of Fe-Ti) is between r(Fe-Ti) and r(Fe-Ti) and this problem is caused by the electronic interaction between metals [32]. In addition, the RDF curve in Figure 9a,b has another peak at r > 2.55 Å and 2.93 Å, except for the first main peak, which indicates that the Fe and Ti crystal structures are ordered. 

The peak values of g(rFe-Fe) and g(rTi-Ti) are much larger than that of g(rFe-Ti), because the bonding strength of Fe-Fe and Ti-Ti is greater than that of Fe-Ti. With the increase in diffusion time, the peak strength of g(rFe-Fe) and g(rTi-Ti) decreases, whereas the peak strength of g(rFe-Ti) increases. It can be explained as follows: during the diffusion process, the atoms in the crystal migrate, resulting in the crystal structure becoming disordered and the bonding strength between the same atoms decreasing. While the bonding reaction between different atoms occurs, the Fe atoms and Ti atoms combine with each other, resulting in an increase in bonding strength. In addition, raising the temperature will also result in (rFe-Fe) and g(rTi-Ti) peak decreases and g(rFe-Ti) peak increases. It indicates that the increase in temperature will accelerate the disorder of crystal structure at the interface and the mutual diffusion of atoms at the interface.

### 3.3. Diffusion Coefficient Analysis

The Einstein method has been used to investigate the atom diffusion behavior at the interface. Firstly, the mean square displacement (MSD) of the atom was calculated. Through the relationship between the MSD curves and the diffusion coefficient, the diffusion coefficient *D* of the particle can be obtained, in which the MSD can be calculated as [33]:

(3) $$MSD=\frac{1}{N}\sum_{i=1}^{N}\langle {\left|r_{i}(t)-r_{0}(0)\right|}^{2}\rangle$$

where *N* is the number of atoms; $r_{i}\left(t\right)$ and $r_{i}\left(0\right)$ are displacement vectors of *i* atom at zero and *t* moment, respectively; the calculated symbol 〈〉 is temporal correlation.

When the running time tends to be infinite:

(4) $$\underset{t\to \infty }{\lim}MSD=c+2dDt$$

where d is the dimension of the system; D is the diffusion coefficient; c is constant. The mean square displacement is listed as a function of time and the slope of the curve is 2dDt. The system discussed in this paper is three-dimensional, so the diffusion coefficient can be expressed as [9]:

(5) $$D=\frac{1}{6}\underset{t\to \infty }{\lim}(\frac{d}{dt}\langle {\left|r_{i}(t)-r_{0}(0)\right|}^{2}\rangle )$$

The temperature was discovered to be a key factor influencing the diffusion rate in the preceding investigation. The general expression of diffusion coefficient can be established by Arrhenius relation [33]:

(6) $$D=D_{0}\exp(-\frac{Q}{RT}),$$

where D is the diffusion coefficient; $D_{0}$ is diffusion factor; Q is the diffusion activation energy; R is the gas constant, R = 8.314 J/(mol K).

Taking the logarithm of Equation (6), the relationship between *D* and *T* could be expressed as:

(7) $$\ln D=\ln D_{0}-\frac{Q}{RT},$$

**Figure 11:**
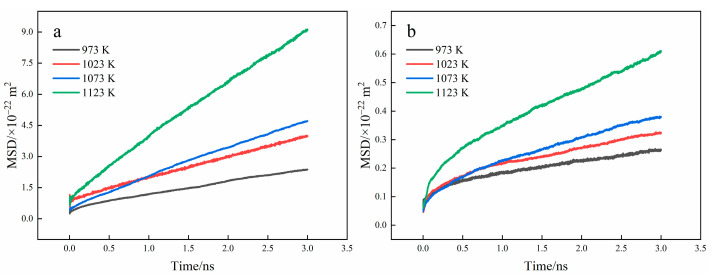
MSD curves of Fe and Ti atoms at different temperature ((**a**) Fe atoms; (**b**) Ti atoms).

The MSD curves of the Fe and Ti atoms were obtained by the MD simulations, illustrated in Figure 11. The diffusion of Fe atoms is relatively stable, while the movement of Ti atoms is relatively intense at the beginning of diffusion and then gradually tends to be stable. Furthermore, the MSD of Fe and Ti atoms gradually increases as temperature rises. 

**Figure 12:**
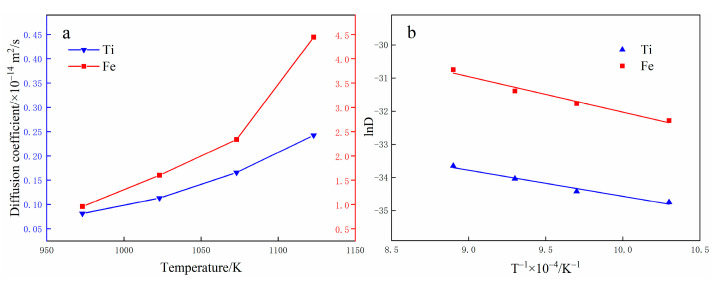
Relationship between diffusion coefficient and temperature.

The diffusion coefficients of Fe and Ti atoms at the interface with different temperatures are calculated according to the MSD curves and Equation (5). It can be found from Figure 12 that the diffusion coefficient is exponentially related to the diffusion temperature, which is consistent with the results of Equation (6) and Equation (7). The D-value of Fe atoms is higher than that of Ti atoms and the D-value of Fe atoms and Ti atoms increases with the rise in temperature. When the diffusion temperature is 973 K, the diffusion coefficients of Fe and Ti atoms are 9.57 × 10^−15^ m^2^/s and 8.12 × 10^−16^ m^2^/s, respectively. In addition, when the diffusion temperature is less than 1073 K, the diffusion coefficients of Fe and Ti atoms are small and the atoms are in the low-speed diffusion stage. When the diffusion temperature is greater than 1073 K, the diffusion coefficient of Fe atoms and Ti atoms increases obviously and the atoms are in the rapid diffusion stage. As the diffusion temperature reaches 1123 K, the diffusion coefficients of iron and titanium atoms reach 4.45 × 10^−14^ m^2^/s and 2.43 × 10^−15^ m^2^/s, respectively. As a result, atomic diffusion behavior occurs mostly at high temperatures, whereas there are no discernible diffusion phenomena at low temperatures. This is because atoms at low temperatures cannot get sufficient energy to break the potential barrier, yet atoms at high temperatures can obtain sufficient kinetic energy.

## 4. Conclusions

The atomic diffusion behavior of the Fe-Ti composite interface at different times and temperature was simulated by using MD simulation. The following conclusions can be obtained:

1. As diffusion time and temperature rise, the boundary between the Fe and Ti layer dissolves, the thickness of the diffusion layer grows, and the diffusion amount of Fe atoms to the Ti layer exceeds that of Ti atoms to the Fe layers.

2. During the diffusion process, the diffusion boundary experienced significant plastic deformation and shear strain. The structure of the diffusion area was disrupted and flaws were formed, which enhanced atom diffusion at the interface. The shear strain of atoms at the contact rose as diffusion time and temperature increased.

3. There is only one primary peak in the RDF curves of Ti and Fe atoms. Both short- and long-range order may be seen in the crystal structure. As the diffusion time and temperature increase, the peak intensity decreases and the order of the crystal structure is destroyed during diffusion.

4. The MSD curves were extracted and utilized to calculate Ti and Fe atom’s diffusion coefficients and the diffusion rate of Fe atoms is greater than that of Ti atoms.

5. As the diffusion temperature rises, the diffusion coefficients and the thickness of the diffusion area in the interaction area increase and there is a linear relationship between the logarithm of the diffusion coefficient

## Data Availability

Not applicable.
